# Supplementary material for: Risk Factors of Long-Term Care Insurance Certification in Japan: A Scoping Review
Source: Int J Environ Res Public Health. 2022 Feb 14;19(4):2162. doi: 10.3390/ijerph19042162 (PMC8872097; doi:10.3390/ijerph19042162)
Supplement: Supplementary file 1 [file ijerph-19-02162-s001.zip › Table_S1_ST1KT1/Table_S1_ST1KT1.pdf]

Table S1. Summary of study characteristics and findings (in the order of citation)

Study	Sample size, Project name†, Subjects' age	Design	Baseline survey year	Follow-up periods	Endpoint (Support/Care-need level)	Exposure variable ‡	Covariates/ confounders ‡	Results *‡
T. Akune, et al. 2014 [12]	1773, ROAD, Aged 65 years or older	Prospective Cohort	2005	4 years	Disability (All levels)	(1) Grip strength (/kg)<br>(2) Knee extension torque (/kg)<br>(3) Usual gait speed (/0.1 m/s)<br>(4) Chair stand time (/s)<br>(5) Muscle dysfunction (yes/no)	Sex, age, smoking, alcohol weight, BMI	Men<br>(1) Grip strength, 0.95 (0.89–0.99)<br>(2) Knee extension torque, 0.97 (0.95–0.99)<br>(3) Usual gait speed, 0.83 (0.74–0.92)<br>(4) Chair stand time, 1.18 (1.10–1.27)<br>(5) Muscle dysfunction, 1.68 (0.91–3.09)<br><br>Women<br>(1) Grip strength, 0.94 (0.89–0.98)<br>(2) Knee extension torque, 0.97 (0.95–1.00)<br>(3) Usual gait speed, 0.85 (0.78–0.92)<br>(4) Chair stand time, 1.06 (1.02–1.09)<br>(5) Muscle dysfunction, 1.72 (1.04–2.85)
H. Otsuka, et al. 2019 [13]	489, The Ogano study conducted in Ogano-machi in Saitama Prefecture, Aged 65 years or older	Prospective Cohort	2014	7.9 years	Disability (All levels)	(1) The timed up and go (TUG) test<br>(2) Hand grip strength (HGS)	Age, BMI, and the follow-up period.	(1) TUG, OR=1.216 (1.074–1.377)<br>(2) HGS, not significant

S. Moriya, et al. 2013 [14]	784, Aged 65 years or older in Tomamae and Iwaai in Hokkaido	Prospective Cohort	2004-2005	5 years	Disability (All levels)	(1) Handgrip strength (grouped by quantiles)<br>(2) One-leg standing time with eye open (OLST) (quantiles)	Age, type of household, socioeconomic status, chronic medical diseases, BMI, the number of natural teeth.	Women<br>(1) Handgrip strength (ref: 25.0–32.0 kg) Not significant<br>(2) OLST (ref: 30.0–120.0 seconds) 0.0–5.9 seconds, 3.7 (1.2–11.2)<br><br>Men<br>(1) Handgrip strength (ref: 38.0–51.0 kg) Not significant<br>(2) OLST (ref: 40.0–120.0 seconds) 0.0–5.9 seconds, 8.6 (1.1–69.2)
K. Makino, et al. 2019 [15]	693, NCGG-SGS, Aged 70 years or older with chronic lower back or knee pain	Prospective Cohort	2015	2 years	Disability (All levels)	(1) Step counts<br>(2) Moderate– to vigorous–intensity physical activity duration<br>(3) Light-intensity physical activity duration	Age, sex, medication, hypertension, diabetes mellitus, heart disease, osteoarthritis, spinal disease, eye disease, MMSE score, GDS score, fall history, severity of pain, site of pain, and gait speed.	(1) Step counts, 1.79 (1.02–3.14)<br>(2) Moderate– to vigorous–intensity physical activity duration, 2.02 (1.16–3.51)<br>(3) Light intensity physical activity duration, 1.72 (0.97–3.05)
T. Akune, et al. 2014 [16]	1773, ROAD, Aged 65 years or older	Prospective Cohort	2005	4 years	Disability (All levels)	The Western Ontario and McMaster Universities Osteoarthritis Index (WOMAC)	Sex, age, BMI, smoking, alcohol, region	Overall population<br>WOMAC ≥4, 2.54 (1.76–3.67)<br><br>Men

								WOMAC ≥5, 1.88 (1.03–3.43)<br><br>Women<br>WOMAC ≥4, 3.13 (1.95–5.02)
Y. Fujiwara, et al. 2006 [17]	1055, Aged 65 years or older	Prospective Cohort	2000	3 years 4 months	Disability (All/Care-need level ≥2)	Not specified (Age, medical history (stroke, heart disease, hypertension, diabetes, arthritis), hospitalization in last year, walking ability, cognitive function (MMSE, only in men), self-rated health, IADL (the Tokyo Metropolitan Institute of Gerontology Index of Competence [TMIG-IC]), spouse (only in men), smoking (only in men), chewing ability (only in women))	Age, medical history (stroke, heart disease, hypertension, diabetes, arthritis), hospitalization in last year, walking ability, cognitive impairment (MMSE, only in men), self-rated health, IADL (the Tokyo Metropolitan Institute of Gerontology Index of Competence [TMIG-IC]), spouse (only in men), smoking (only in men), chewing ability (only in women)	All levels<br>Men<br>No spouse, 5.01 (1.15–21.73)<br>Smoking, 5.59 (1.31–23.76)<br>Poor walking ability, 7.22 (1.56–33.52)<br><br>Women<br>Poor walking ability, 3.28 (1.28–8.42)<br>Hospitalization in last year, 4.02 (1.44–11.20)<br>Poor chewing ability, 4.42 (1.86– 10.54)<br><br>Care–need level ≥2<br>Men<br>Poor IADL (TMIG–IC≤4), 3.74 (1.59–8.76)<br>Severe cognitive decline, 4.94 (1.70–14.36)<br><br>Women

								Poor IADL (TMIG–IC<=4), 3.90 (1.32–11.54).
H. Hirai, et al. 2009 [18]	9702, JAGES, Aged 65 years and older	Prospective Cohort	2003	3 years	Disability (All/Care-need level ≥2)	Not specified (Age, family structure, socioeconomic status, diseases under treatment, number of medications, falls in 1 year, biting ability, BMI, hearing impairment, vision impairment, excretion impairment, the Tokyo Metoroplitan Institute of Gerontology index of competence (TMIG-IC), depression (GDS), self-rated health, alcohol consumption, smoking, walking time in a day, frequency of going out, frequency of contact with friends, social support, participation in group activity, waking and housekeeping)	Age, family structure, socioeconomic status, diseases under treatment, number of medications, falls in 1 year, biting ability, BMI, hearing impairment, vision impairment, excretion impairment, the TMIG-IC, depression, self-rated health, alcohol consumption, smoking, walking time in a day, frequency of going out, frequency of contact with friends, social support, participation in group activity, waking and housekeeping	Men<br>Family structure (ref: living only with spouse)<br>Living alone, 1.94 (1.25–3.01)<br>Others, 1.63 (1.14–2.33)<br>Years of education attainment (ref: ≥13 years)<br>6–9 years, 1.60 (1.11–2.33)<br><6 years, 1.62 (1.50–4.60)<br>Diseases under treatment, 1.77 (1.35–2.31)<br>Falls in 1 year, 2.46 (1.81–3.34)<br>Poor biting ability, 1.63 (1.18–2.25)<br>BMI (ref: ≥18.5 kg/m^2)<br><18.5, 1.44 (1.04–1.99)<br>Excretion impairment, 1.50 (1.13–1.98)<br>TMIG–IC score (ref: 13 points)<br>0–12, 1.73 (1.35–2.22)<br>Disability of instrumental activity of daily living, 1.94 (1.54–2.44)<br>Intellectual disability, 1.79 (1.43–2.25)<br>Social role disability, 1.44 (1.14–1.81)<br>Poor self- rated health, 2.79 (2.25–3.47)

								Depression (ref: no depression)<br>Depressive tendency, 1.65 (1.26–2.15)<br>Depressive state 1.98 (1.32–2.98)<br>Non-alcohol drinking, 1.42 (1.14 –1.77)<br>Walking time per a day (ref: ≥30 minutes)<br><30 minutes, 1.66 (1.33–2.07)<br>Frequency of going out (ref: almost every day)<br>≤1 time/week, 1.62 (1.24–2.11)<br>Frequency of contact with friends (ref: 1 time/month)<br>< 1 time/month, 1.45 (1.16–1.81)<br>Non-participation in voluntary group activity, 1.33 (1.03–1.73)<br>No working, 1.75 (1.30–2.35)<br>No housekeeping, 1.33 (1.07–1.65)<br><br>Women<br>Diseases under treatment, 1.29 (1.05–1.60)<br>Falls in 1 year, 1.83 (1.45 –2.31)<br>Poor biting ability, 1.73 (1.34–2.23)<br>Excretion impairment, 1.66 (1.29–2.14)<br>TMIG-IC score (ref: 13 points)<br>0–12 points, 1.91 (1.53– 2.39)

								Disability of instrumental activity of daily living, 1.84 (1.49–2.26)<br>Intellectual disability, 1.52 (1.26–1.83)<br>Social role disability, 1.69 (1.39–2.05)<br>Poor self-rated health, 2.28 (1.91–2.73)<br>Depression (ref: no depression)<br>Depressive tendency, 1.82 (1.46–2.28)<br>Depressive state, 3.30 (2.48–4.40)<br>Current smoking, 2.11 (1.30–3.43)<br>Walking time per a day (ref: ≥30 minutes)<br>< 30 minutes, 1.29 (1.07–1.56)<br>Frequency of going out (ref: almost every day)<br>≤1 time/week, 1.46 (1.17–1.83)<br>Frequency of contact with friends (ref: 1 time/month)<br>< 1 time/month, 1.29 (1.05–1.59)<br>Non-emotional support, 1.54 (1.15–2.06)<br>Non–participation in regular group activity, 1.30 (1.06–1.58)<br>Non-participation in voluntary group activity, 2.17 (1.66–2.85)<br>No working, 1.81 (1.24–2.64)<br>No housekeeping, 1.85 (1.49–2.30)

K. Tsutsumimoto, et al. 2016 [19]	4038, OSHPE, Aged 65 years or older without Parkinson diseases	Prospective Cohort	2011-2012	29-32 months	Disability (All levels)	Interaction of walking speed <1.0m/s defined as slow gait speed (a 2.4m walking path) and Depression (GDS ≥6)	Age, sex, socioeconomic status, hypertension, diabetes mellitus, hyperlipidemia, ear disease, osteoarthritis, medication, pain, MMSE, physical activity, sleeping time	(Ref: slow gait speed (-) or depressive symptom (-))<br>Slow gait speed (+) and depressive symptom (+), 3.08 (2.00−4.75)<br>Slow gait speed (+), 2.44 (1.71−3.47)<br>Depressive symptom (+), 1.60 (1.01−2.53)
M. Hoshi, et al. 2012 [20]	823, The Tsurugaya project, Aged 70 years or older	Prospective Cohort	2003	4 years	Disability (All levels)	Motor Fitness Scale (MFS)	Age, height, weight, BMI, maximum walking velocity, time up & go test (TUG), leg extension power, functional reach test	(Ref: MFS of 14)<br>MFS 12 for men (10–12 for women), 2.05 (1.20–3.50)<br>MFS 0–11 for men (0–9 for women), 3.04 (1.80–5.12)<br><br>Stratified by MMSE<br>MMSE score 28–30 and 26–27, a lower MFS quartile had a significantly increased risk of LTC.
S. Chen, et al. 2019 [21]	1554, The Sasaguri Genkimon Study (SGS), Aged 65 years or older	Prospective Cohort	2017	5.8 years	Disability (All levels)	(1) Cardiovascular Health Study (CHS) criteria<br>(2) A simple frailty questionnaire (FRAIL)	Age, sex, socioeconomic status, living alone, household finance, smoking, alcohol, cognitive impairment, polypharmacy	(1) CHS criteria (ref: robust)<br>Prefrail, 1.50 (1.10–2.03)<br>Frail, 2.00 (1.32–3.02)<br>(*p* for trend =0.001)<br><br>(2) The FRAIL scale (ref: robust)

								Prefrail, 1.73 (1.28–2.35)<br>Frail, 2.11 (1.25–3.56)<br>(*p* for trend <0.001)
H. Makizako, et al. 2015 [22]	4341, OSHPE, Aged 65 years or older excluded without Parkinson diseases	Prospective Cohort	2011-2012	2 years	Disability (All levels)	Number of applicable items in following conditions: slowness, hand grip strength, exhaustion, physical activity, and weight loss.<br>Classification<br>robust (0 items), prefrail (1or 2 items), and frail (≥3 items)	Age, sex, BMI, MMSE, prescribed medications (number), medical history (hypertension, heart disease, diabetes mellitus, osteoporosis), GDS	(Ref: robust)<br>Prefrail, 2.52 (1.56–4.07)<br>Frail, 4.65 (2.63–8.22)
H. Shimada, et al. 2015 [23]	5104, OSHPE, Aged 65 years or older without Parkinson diseases	Prospective Cohort	2011-2012	2 years	Disability (All levels)	Frail estimated by Fried's original studies<br>0: non-frail<br>1-2: prefrail<br>3+: frail<br>with/without slow walking speed	Age, sex, BMI>=27.5, <18.5, knee pain, heart disease, pulmonary disease, osteoarthritis, diabetes, MMSE, GDS-15, ADL	(Ref: non-frail)<br>Prefrail without slow walking speed, 1.86 (1.19−2.92)<br>Prefrail with slow walking speed, 3.62 (2.19−5.96)<br>Frail without slow walking speed, 4.33 (2.00−9.39)<br>Frail with slow waling speed, 4.68 (2.72−8.05)

T. Tanaka, et al. 2017 [24]	1212, Aged 65 years old and over living in Kawasaki City	Prospective Cohort	2012	4 years	Disability (Care-need level ≥3)	Yubi-wakka (finger ring) test (comparison between finger-ring and non-dominant calf circumference)<br>Calf circumference is Bigger/ Just fits/ Smaller	Age, socioeconomic status, no physical activity, chronic diseases (low nutrition, high blood pressure, diabetes, hyperlipidemia) (osteoporosis, cancer, heart disease), calf pedal edema, and body fat percentage	Total (ref: bigger)<br>Just fits 1.11 (0.73–1.68)<br>Smaller 1.96 (1.68–5.93)<br><br>Male (ref: bigger)<br>Just fits 1.09 (0.58–2.06)<br>Smaller 3.55 (1.91–6.59)<br><br>Female (ref: bigger)<br>Just fits 1.05 (0.50–2.21)<br>Smaller 1.21 (0.67–2.11)
S. Moriya, et al. 2012 [25]	812, Aged 65 years or older in Tomamae and Iwaai in Hokkaido	Prospective Cohort	2004-2005	5 years	Disability (All levels)	Self-assessed masticatory ability (3 categories: ability to chew all foods (good masticatory ability), ability to chew only slightly hard food (fair masticatory ability), and ability to only chew soft or pureed food (poor masticatory ability)	Age, sex, socioeconomic status, chronic medical conditions, dentition status	(Ref: good)<br>Fair/poor, 1.87 (1.07–3.27)
T. Matsunaga, et al. 2017 [26]	2888, NISSIN, Aged 64 or 65 years old in the Nisshin city	Prospective Cohort	2013	11.6 years	Disability (All/Care-need level ≥2),	Leisure-time physical activity (tertile,0 METS-hours/week, 0.1 -18	Year of participation, work, marital status, educational attainment, smoking, drinking, BMI,	Dementia<br>Men (ref: no activity)<br>≥18.1 MET hrs/wk, 0.48 (0.2−0.94)

					Dementia, Disability (Care-need level ≥2) or Death	METS-hour/week, 18.1-261.9 METS-hours/week)	and comorbidity (hypertension, diabetes mellitus dyslipidemia, and neuralgia and/or low back pain), GDS score, social activity, and total walking time/day	Women<br>Not significant
Y. Tomata, et al. 2017 [27]	6909, The Ohsaki Cohort 2006, Aged 65 years or older	Prospective Cohort	1994 and 2006	5.7 years	Dementia	Change in amount of time spent walking per day between 1994 to 2006	Age, sex, BMI, medical history (stroke, hypertension, myocardial infarction, diabetes or hyperlipidemia), smoking, alcohol, psychological distress, pain, physical functioning level (in 1994)	(Ref: <0.5 hr (1994)→<0.5 hr (2006))<br>0.5-1 hr → <0.5 hr, 1.01 (0.74−1.37)<br>≥1 hr → <0.5 hr, 1.08 (0.80−1.46)<br><0.5 hr → 0.5-1 hr, 0.92 (0.65−1.30)<br>0.5-1 hr → 0.5-1 hr, 0.80 (0.59−1.10)<br>≥1 hr → 0.5-1 hr, 0.92 (0.65−1.30)<br><0.5 hr → ≥1 hr, 1.12 (0.70−1.80)<br>0.5 -1 hr → ≥1 hr, 1.05 (0.72−1.55)<br>≥1 hr → ≥1 hr, 0.72 (0.53−0.97)
Y, Tomata, et al. 2019 [28]	13390, The Ohsaki Cohort 2006, Aged 65 years or older	Prospective Cohort	2006	5.7 years	Dementia	Time spent walking daily	Age, sex, BMI, education, smoking, alcohol, psychological distress, medical history (stroke, hypertension, myocardial infarction, diabetes, and dyslipidemia)	(Ref: <0.5 hr/day)<br>0.5− 1 hr/day, 0.81 (0.71−0.92)<br>≥1 hr/day, 0.72 (0.62−0.84)

K. Makino, et al. 2017 [29]	4329, OSHPE, Aged 65 years or older	Prospective Cohort	2011-2012	52 months	Disability (All levels)	Combination of fall history and fear of falling (FOF)	Age, sex, number of tablets, medical history (hypertension, diabetes, heart disease, ankylosing knee arthritis), walking speed, cognition function (MMSE), depression (GDS)	(Ref: Fall (-) & FOF (-))<br>Fall (+) & FOF (-), 1.41 (0.96−2.08)<br>Fall (-) & FOF (+), 1.28 (1.01−1.62)<br>Fall (+) & FOF (+), 1.44 (1.05−1.98)
A. Ikeda, et al. 2008 [30]	Case 208, Control 416, Aged 65 years old or older between 2000 and 2004 in Ikawa town	Nested case-control	1981 and 1994	6-23 years	Dementia	(1) Smoking status: Current smoker(<20 cigarettes/day, ≥ 20 cigarettes/day), past smoker, and never smoker<br>(2) Number of years of smoking (never smoker, <34 years, 34 to <45 years, ≥45 years<br>(3) Pack-years of smoking (<28, 28 to <46, ≥46)	SBP, alcohol, TC, use of antihypertensive medication, diabetes mellitus, atrial fibrillation and ST-T abnormality	(1) Smoking status (ref: never smoker)<br>Current smoker, OR=2.3 (1.1−4.7)<br><br>(2) Years of smoking (ref: never smokers)<br>≥45, OR=2.3 (1.0−5.4)<br>(*p* for trend=0.04).<br><br>(3) Pack-years of smoking<br>Not significant
M, Noguchi-Shinohara, et al. 2019 [31]	2647, Aged 65 years and older	Prospective Cohort	2006	10 years	Dementia	Smoking and diabetes mellitus	Age, sex, SBP, TC, ECG abnormality, alcohol	Interaction between current smoking & diabetes, 1.85 (1.39−2.47)<br><br>(Ref: never smoking & diabetes (-))

								Former smoking & diabetes (-), 1.04 (0.98−1.21)<br>Current smoking & diabetes (-), 1.25 (1.05−1.48)<br>Never smoking & diabetes (+), 1.31 (1.16−1.47)<br>Former smoking & diabetes (+), 1.26 (0.95−0.65)<br>Current smoking & diabetes (+), 1.86 (1.39−2.48)
Y. Tomata, et al. 2012 [32]	13988, The Ohsaki Cohort 2006, Aged 65 years or older	Prospective Cohort	2006	3 years	Disability (All levels)	Consumption of<br>(1) Green tea<br>(2) Oolong tea<br>(3) Black tea<br>(4) Coffee	Age, sex, medical history (stroke, myocardial infarction, hypertension, arthritis, osteoporosis, or fracture), socioeconomic status, smoking, alcohol, BMI, cognitive activity score, psychological distress, time spent walking, consumption volume of rice, miso soup, meat, fish, green and yellow vegetables, potatoes, soy products,	(1) Green tea (ref: <1 cup/day)<br>1−2 cups/day, 0.90 (0.77−1.06)<br>3−4 cups/day, 0.75 (0.64−0.88)<br>≥5 cups/day, 0.67 (0.57−0.79)<br><br>(2) Oolong tea (ref: <1 cup/day)<br>1−2 cups/day, 1.47 (1.07−2.03)<br>3−4 cups/day, 0.77 (0.42−1.40)<br>≥5 cups/day, 1.25 (0.71−2.18)<br><br>(3) Black tea (ref: <1 cup/day)<br>1−2 cups/day, 1.23 (0.96−1.59)<br>3−4 cups/day, 0.82 (0.45−1.51)<br>≥5 cups/day, 1.01 (0.74−1.41)

							fruits and sweets, social support, participation in community activities, and motor function score	(4) Coffee (ref: <1 cup/day)<br>1−2 cups/day, 0.90 (0.79−1.03)<br>3−4 cups/day, 0.93 (0.72−1.22)<br>≥5 cups/day, 1.02 (0.74−1.41)
S. Matsuyama, et al. 2019 [33]	2923, The Ohsaki Cohort 2006, Aged 65 years or older	Prospective Cohort	1994 and 2006	7.7 years	Disability (All levels)	Change in the Japanese Diet Index (JDI) between 1994 and 2006	Age, sex, BMI, smoking, alcohol, time spent walking, energy intake, history of disease (hypertension, diabetes, myocardial infarction, or stroke), socioeconomic status, psychological distress (K6)	The JDI score (ref: ≤ -2)<br>≤ -1, 0.82 (0.66−1.01)<br>No change, 0.80 (0.65−0.98)<br>≥ +1, 0.76 (0.61−0.95)<br>≥ +2, 0.77 (0.61−0.98)
Y. Tomata, et al. 2014 [34]	14260, The Ohsaki Cohort 2006, Aged 65 years or older	Prospective Cohort	2006	5 years	Disability (All levels)	Groups divided by quantiles of three dietary patterns scores derived using principal component analysis of 39 food and beverage items of food frequency questionnaire<br>(1) Japanese pattern<br>(2) Animal food pattern<br>(3) High dairy pattern	Age, sex, smoking, alcohol, BMI, socioeconomic status, medical history (stroke myocardial infarction, hypertension, arthritis, osteoporosis fracture) psychological distress, time spent walking, motor	(1) Japanese pattern (ref: Q1)<br>Q2, 0.91 (0.82–1.02)<br>Q3, 0.82 (0.73–0.92)<br>Q4, 0.77 (0.68–0.88)<br>(*p* for trend <0.001)<br><br>(2) Animal food pattern (ref: Q1)<br>Q2, 1.10 (0.98–1.23)<br>Q3, 1.07 (0.95–1.21)<br>Q4, 1.16 (1.02–1.31)

							function score, total energy intake, protein intake	(*p* for trend = 0.053)<br><br>(3) High dairy pattern (ref: Q1)<br>Q2, 0.99 (0.88–1.11)<br>Q3, 0.95 (0.84–1.07)<br>Q4, 1.11 (0.99–1.26)<br>(*p* for trend = 0.158)
K. Sugiyama, et al. 2016 [35]	13137, The Ohsaki Cohort 2006, Aged 65 years or older	Prospective Cohort	2006	5.7 years	Dementia	Coffee consumption	Age, sex, BMI, medical history (stroke, hypertension, diabetes mellitus, myocardial infarction, arthritis, osteoporosis, and facture), smoking, alcohol, green tea consumption, socioeconomic status, walking duration psychological distress score, perception of feelings for social support, participation in community activities	(Ref: never)<br>Occasionally, 0.73 (0.62–0.86)<br>1–2 cup/day, 0.72 (0.61–0.84)<br>≥3 cup/day, 0.82 (0.65–1.02)

Y. Tomata, et al. 2016 [36]	13645, The Ohsaki Cohort 2006, Aged 65 years or older	Prospective Cohort	2006	5.7 years	Dementia	Consumption of<br>(1) Green tea<br>(2) Oolong tea<br>(3) Black tea<br>(4) Coffee	Age, sex, BMI, medical history (stroke, hypertension, diabetes mellitus, myocardial infarction, arthritis, osteoporosis, and facture), smoking, alcohol, socioeconomic status, walking duration psychological distress score, perception of feelings for social support, participation in community activities, motor function score, consumption volume of specific foods (green and yellow vegetables and fruit), coffee consumption, and energy intake	(1) Green tea (ref: <1 cup/day)<br>1−2 cups/day, 1.06 (0.89−1.27)<br>3−4 cups/d, 0.88 (0.74−1.04)<br>≥5 cups/d, 0.73 (0.61−0.87)<br><br>(2) Oolong tea (ref: <1 cup/day)<br>1−2 cups/day, 0.69 (0.46−1.04)<br>3−4 cups/day, 1.03 (0.62−1.72)<br>≥5 cups/day, 0.68 (0.34−1.35)<br><br>(3) Black tea (ref: <1 cup/day)<br>1−2 cups/day, 1.05 (0.80−1.39)<br>3−4 cups/day, 1.13 (0.68−1.87)<br>≥5 cups/day, 0.71 (0.28−1.79)<br><br>(4) Coffee (ref: <1 cup/day)<br>1−2 cups/day, 0.90 (0.79−1.03)<br>3−4 cups/day, 0.93 (0.72−1.22)<br>≥5 cups/day, 1.02 (0.74−1.41)
Y. Tomata, et al. 2016 [37]	14402, The Ohsaki Cohort 2006, Aged 65 years or older	Prospective Cohort	2006	4.9 years (S.D. 1.5)	Dementia	Groups divided by quantiles of three dietary patterns scores derived using principal component analysis of 39 food and	Age, sex, smoking, alcohol, BMI, medical history (stroke myocardial infarction, hypertension, arthritis, osteoporosis	(1) Japanese pattern (ref: Q1)<br>Q2, 0.95 (0.81–1.11)<br>Q3, 0.85 (0.71–1.01)<br>Q4, 0.80 (0.66–0.97)<br>(*p* for trend = 0.016)

						beverage items of food frequency questionnaire<br>(1) Japanese pattern<br>(2) Animal food pattern<br>(3) High dairy pattern	fracture) psychological distress, time spent walking, Kihon-check list (motor function score, and cognitive function score), number remaining teeth, total energy intake, protein intake, socioeconomic status	(2) Animal food pattern (ref: Q1)<br>Q2, 1.09 (0.93–1.28)<br>Q3, 1.13 (0.95–1.33)<br>Q4, 1.12 (0.92–1.36)<br>(*p* for trend = 0.216)<br><br>(3) High dairy pattern (ref: Q1)<br>Q2, 0.88 (0.76–1.03)<br>Q3, 0.99 (0.84–1.16)<br>Q4, 0.97 (0.83–1.15)<br>(*p* for trend = 0.896)
S. Zhang, et al. 2017 [38]	13373, The Ohsaki Cohort 2006, Aged 65 years or older	Prospective Cohort	2006	5.7 years	Dementia	The frequency of citrus intake	Age, sex, BMI, medical history (stroke, hypertension, myocardial infarction, and diabetes), socioeconomic status, smoking, alcohol, psychological distress, time spent walking, motor function score, intake (total vegetables, other fruits, protein, and energy)	(Ref: ≤2 times/week)<br>3–4 times/week, 0.92 (0.80–1.07)<br>Almost every day, 0.86 (0.73–1.01)<br>(*p* for trend = 0.065)

S. Zhang, et al. 2017 [39]	13230, The Ohsaki Cohort 2006, Aged 65 years or older	Prospective Cohort	2006	5.7 years	Dementia	Mushroom consumption	Age, sex, BMI, medical history (stroke, hypertension, myocardial infarction, diabetes, or hyperlipidemia), socioeconomic status, smoking, alcohol, psychological distress, time spent walking, motor function score, Intake (consumption volume of meat, fish, green and yellow vegetables, and fruits)	(Ref: <1 time/week)<br>1–2 times/week, 0.95 (0.81–1.10)<br>≥3 times/week, 0.81 (0.69–0.95)<br>(*p* for trend <0.01)
Y. Lu, et al. 2018 [40]	7422, The Ohsaki Cohort 2006, Aged 65 years or older	Prospective Cohort	1994 and 2006	5.7 years	Dementia	The change of sleep duration between 1994 and 2006.	Age, sex, BMI, socioeconomic status, smoking, alcohol, time spent on walking, pain, psychological distress, history of diseases (stroke, hypertension, myocardial infarction, diabetes, and desideria)	(Ref: no change in sleep duration)<br>Decreased by ≥2 hours, 1.02 (0.72−1.43)<br>Decreased by 1 hour, 1.05 (0.82–1.36)<br>Increased by 1 hour, 1.31 (1.07–1.60)<br>Increased by ≥2 hours, 2.01 (1.51 to 2.69)

L. Zhang, et al. 2019 [41]	1895, NISSIN, Aged 64 or 65 years old in the Nisshin city	Prospective Cohort	2005	12.7 years	Disability (All levels)	Sleep duration (<6, 6–6.9, 7–7.9, 8–8.9 and ≥9 h/day)	BMI, socioeconomic status, working status, marital status, smoking, alcohol, walking status, depressive tendency, history of diseases (cardiovascular disease, cancer, diabetes, hypertension, hyperlipidemia)	(Ref: 7–7.9 h/day)<br>Total<br><6 h/day, 1.64 (1.13–2.38)<br><br>Men<br>Not significant<br><br>Women<br><6 h/day, 1.90 (1.19–3.03)
S. Zhang, et al. 2018 [42]	9910, The Ohsaki Cohort 2006, Aged 65 year or older	Prospective Cohort	2006	10 years	Disability (All levels)	Healthy life index (HLI, 0 to 3) (summed number of Healthy Lifestyle Behaviors ('never or former smoker', 'time spent walking ≥0.5 hour/day' and 'vegetable and fruit consumption volume ≥median'))	BMI, medical history, education, social support, motor function score (the Kihon Check list), cognitive function score (the Kihon Check list)	HLI score (one point increments), 0.85 (0.81–0.90)
S. Zhang, et al. 2018 [43]	9910, The Ohsaki Cohort 2006, Aged 65 years or older	Prospective Cohort	2006	10 years	Disability (All/Care-need level ≥2)	Healthy lifestyle Index (HLI, 0 to 3)	Age, sex, BMI, presence of chronic conditions, socioeconomic status, social support situation,	HLI score (ref: 0 and 1)<br>All levels<br>HLI score 2, 0.78 (0.73–0.84)<br>HLI score 3, 0.71 (0.65–0.77)

							motor function status, cognitive impairment	Care need level ≥2 or death<br>HLI score 2, 0.73 (0.68–0.79)<br>HLI score 3, 0.63 (0.56–0.70)
A. Yagi, et al. 2019 [44]	13786, JAGES, aged 65 years or older	Prospective Cohort	2010-2012	3 years	Disability (All levels)	Tub bathing frequency in<br>(1) Summer<br>(2) Winter	Age, sex, smoking, drinking, marital status, employment, socioeconomic status, BMI, treatment for any disease, physical strength, cognitive impairment, depressive symptom, and instrumental ADL	Frequency of tub bathing (ref: 0–2 times/week)<br>(1) Summer<br>3–6 times/week, 0.91 (0.75–1.10)<br>≥7 times/week, 0.72 (0.60–0.85)<br><br>(2) Winter<br>3–6 times/week, 0.90 (0.76–1.07)<br>≥7 times/week, 0.71 (0.60–0.84)
J. Aida, et al. 2012 [45]	4425, AGES, Aged 65 years or older	Prospective Cohort	2003	4 years	Disability (All levels)	(1) The number of remaining teeth<br>(2) Self-reported eating ability	Sex, age, BMI, smoking, alcohol, self–rated health, present illness, waking minutes/d, socioeconomic status	(1) The number of teeth (ref: ≥20)<br>≤19, 1.21 (1.06–1.40)<br><br>(2) Self–reported eating ability<br>not significant
T. Komiyama, et al. 2014 [46]	834, The Tsurugaya project, Aged 65 years or older	Prospective Cohort	2003	6.2 years	Disability (All levels)	Presence of a regular dentist	Age, sex, BMI, medical history, smoking, alcohol, socioeconomic status, dietary status, cognitive impairment, depressive symptom, physical	(Ref: had a regular dentist)<br>Didn't have a family dentist, 1.38 (1.02–1.55)

							function, sporting goods, current tooth size	
T. Komiyama, et al. 2016 [47]	834, The Tsurugaya Project, Aged 70 years or older	Prospective Cohort	2002-2003	7.9 years	Disability (All levels)	(1) The number of remaining teeth (≥20, 10−19, 1−9, 0)<br>(2) An assessment of regularity of receiving dental care (routine examinations, preventing oral disease, maintaining oral health after treatment)	Age, sex, BMI, Hypertension, medical history (stroke, myocardial infarction, joint disease), smoking, alcohol, socioeconomic status, depressive symptoms, cognitive impairment (MMSE), physical function, restricted social support, and regular dental care	(1) The number of remaining teeth (ref: 20 remaining teeth)<br>10−19, 1.42 (1.03–1.94)<br>1−9, 1.46 (1.04–2.03)<br>0, 1.49 (1.03–2.14)<br><br>(2) No regularity of receiving dental care, 0.92 (0.67–1.26)<br><br>Interaction (ref: 20 remaining teeth)<br>0-19 remaining teeth with regular dental care, 1.31 (0.82–2.03)<br>0-19 remaining teeth without regular dental care; 1.46 (1.11–1.92)
T. Komiyama, et al. 2018 [48]	838, The Tsurugaya Project, Aged 70 years or older	Prospective Cohort	2011	7.8 years	Disability (All levels)	The status of dentition on the basis of numbers of remaining teeth and occlusal supports (Miyachi's Triangular Classification): Zone A, ≥10 occlusal supports; Zone B, 5-9 occlusal	Age, sex, BMI, medical history (hypertension stroke, myocardial infarction), smoking, alcohol, socioeconomic status, depressive symptom, cognitive impairment, physical	Certification before 3 years from baseline<br>Not significant<br><br>Certification after 3 years from baseline (ref: Zone A)<br>Zones C, 1.98 (1.26–3.11)<br>Zone D, 2.50 (1.54–4.05)<br>(*p* for trend =0.002)

						supports; Zone D, ≤4 occlusal supports and ≥11 remaining teeth and Zone C, ≤10 remaining teeth	function, social support, history of all, and subjective masticatory ability	
T, Ohi, et al. 2019 [49]	815, The Tsurugaya Project, Aged 70 years or older	Prospective Cohort	2002-2003	7.9 years	Disability (All levels)	Bilateral maximum occlusal force using a horseshoe-shaped pressure-indicating film (quartiles)	Age, sex, BMI, hypertension, medical history (stroke, heart diseases, hypertension), smoking, alcohol, education, depressive symptoms, cognitive impairment, physical function, marital status, history of falls, and the number of remaining teeth.	(Ref: Q4)<br>Q3, 1.53 (1.02–2.33)<br>Q2, 1.64 (1.06–2.55)<br>Q1, 1.64 (1.01–2.68)<br>(*p* for trend = 0.011)
S. Bando, et al. 2017 [50]	12370, The Ohsaki Cohort 2006, Aged 65 years or older	Prospective Cohort	2006	5.7 years	Disability (All levels)	The number of teeth (0-9 teeth, 10-19 teeth, 20 or more teeth)	Smoking, alcohol, BMI, time spent walking/d, medical history (stroke, hypertension, myocardial infarction, diabetes mellitus), energy intake, and protein intake	(Ref: ≥20 teeth)<br>10–19 teeth, 1.15 (1.01–1.30)<br>0–9 teeth, 1.20 (1.07–1.34)<br><br>Brushed their teeth <2 times/day with 10-19 teeth, 1.32 (1.12-1.55)<br>Brushed their teeth <2 times/day with 0-9 teeth, 1.32 (1.12-1.55)

T. Yamamoto, et al. 2012 [51]	4425, AGES, Aged 65 years or older	Prospective Cohort	2003-2007	4 years	Dementia	(1) Dental status<br>(2) Mastication<br>(3) Presence of regular dentist<br>(4) Taking care of dental health	Age, sex, alcohol, socioeconomic status, BMI, current illness, exercise, forgetfulness	(1) Dental status (ref: ≥20 teeth)<br>Few teeth with dentures, 1.85 (1.04–3.31)<br><br>(2) Mastication (ref: can chew anything)<br>Cannot chew very well, 1.25 (0.81–1.93)<br><br>(3) Presence of regular dentist (ref: yes)<br>No, 1.44 (1.04–2.01)<br><br>(4) Taking care of dental health (ref: yes)<br>No, 1.76 (0.96–3.20)
A. Nitta, et al. 2010 [52]	783, The Tsurugaya Project, Aged 70 years or older	Prospective Cohort	2003	5 years	Disability (All levels), Disability (All levels) & Death	Presence of peripheral arterial disease (PAD)	Age, sex, smoking, hypertension, high blood glucose, history of stroke, physical activity, functional measures (knee strength extension, functional reach ten-meter maximum walk test, time up go test)	(Ref: no PAD)<br>Disability<br>PAD (+), 1.63 (0.92–2.86)<br><br>Disability & death<br>PAD (+), 1.67 (1.01–2.76)
H. Jinnouchi, et al. 2017 [53]	Case 351, Control 702, CIRCS, Aged 40-89 years old in Ikawa town	Nested case-control	1983 and 2004	6-23 years	Dementia	(1) Retinal vascular changes (generalized arteriolar narrowing, focal arteriolar narrowing,	Sex, age, BMI, overweight, SBP, DBP, hypertension, medication, hyperglycemia, TC,	(1) Retinal vascular changes (ref: absent)<br>Generalized arteriolar narrowing, 1.48 (1.04–2.10)

						arteriovenous nicking, increased arteriolar wall reflex, and any other retinopathy)<br>(2) The number of retinal abnormalities (0, 1, or ≥2)	hypercholesterolemia, smoking, alcohol, incidence of stroke	(2) The number of retinal abnormalities (ref: absent)<br>≥2 retinal abnormalities, 1.50 (1.02–2.22)
M. Yamada, et al. 2013 [54]	8063, J-MACC, Aged 65 years or older	Prospective Cohort	2009	2 years	Disability (All levels)	eGFR ([ml/min/1.73m^2], Modification of Diet in Renal Disease (MDRD) equation arranged Japanese)<br>(Q1, <60.0; Q2, 60.0-71.3; Q3, 71.4-83.6; Q4, >83.6)	Age, sex, BMI, frailty checklist, serum albumin	(Ref: Q4)<br>Q1, 1.63 (1.26–2.09)
S. Lee, et al. 2019 [55]	4457, Aged 65 years or older	Prospective Cohort	2011-2012	4 years	Disability (All levels)	eGFR<br>Stratified by sedentary time (<8 hours/day, ≥8 hours/day)	Age, sex, socioeconomic status, BMI, MMSE score, GDS, smoking, alcohol, medication, total protein, triglyceride, grip strength, gait speed, regular exercise, hypertension, heart disease, osteoporosis, diabetes and cancer.	(Ref: eGFR ≥60 ml/min/1.73m^2)<br>eGFR 45–49, 1.013 (0.798–1.287)<br>eGFR <45, 1.741 (1.193–2.539)<br><br>Sedentary time <8 hr/day<br>eGFR 45–49, 0.941 (0.77–1.31)<br>eGFR <45, 1.447 (0.938–2.234)<br><br>Sedentary time ≥8 hr/day<br>eGFR 45–49, 1.339 (0.824–2.175)

								eGFR <45, 4.367 (2.021–9.428)
T. Himeno, et al. 2017 [56]	1078, Aged 65-94 years old	Prospective Cohort	2008	5 years	Disability (All, due to arthralgia/fractures, due to stroke, due to dementia, due to other disease)	(1) SBP (<130, 130-159, >160)<br>(2) DBP (<80, 80-89, >90) stratified by presence of CKD	Hypertension, diabetes mellitus, hyperuricemia, dyslipidemia, hypoalbuminemia.	Disability<br>(Ref: CKD and SBP of <130)<br>CKD and SBP 130–159, 0.44 (0.26–0.72)<br><br>(Ref; CKD and SBP of 130–159)<br>CKD and SBP of <130, 2.24 (1.48–4.12)<br>non–CKD and SBP of 130–159, 1.58 (1.01–2.52)<br>non–CKD and SBP of <130, 2.25 (1.22–4.13)<br><br>Disability with dementia<br>(Ref: CKD and SBP of <130)<br>CKD (+) and SBP of 130–159, 0.17 (0.05–0.55)<br><br>Interaction CKD and SBP of <130 aged 75 years or older, 0.47 (0.28–0.81)<br>Interaction CKD and SBP of <130 in male, 0.23 (0.09–0.58)
K. Watanabe, et al. 2017 [57]	1078, Aged 65 years or older	Prospective Cohort	2008	5 years	Disability (All levels)	Interaction of presence of CKD (eGFR<60 or dipstick + or more),	Age, sex, living alone, BMI, medical history (hypertension,	(Ref: CKD (-) & DM (-))<br>CKD (+) & DM (+), 2.20 (1.34−3.61)

						Diabetes mellitus (DM) (fasting blood glucose≥ 200mg/dL, HbA1c≥ 6.5%, hypoglycemic agents and/or insulin)	hyperuricemia, hypoalbuminemia)	CKD (+) & DM (+) & HbA1c <6%, 3.52 (1.91−6.48)<br><br>With use of anti-diabetic agents<br>(ref: CKD (+) & DM (+) & HbA1c ≥6%)<br>CKD (+) & DM (+) & HbA1c <6%, 2.40 (1.06−6.45)
S. Nishiguchi, et al. 2013 [58]	5765, J-MACC, Aged 65 years or older in Maibara city	Prospective Cohort	2011	1.5 years	Disability (All levels)	Cognitive Performance Scale; score 0, score 1, and score 2-6	Age, sex, BMI, the number of medications taken, family structure, subjective household economic status, medical history	CPS score (ref: 0)<br>Score 1, 1.39 (1.08–1.77)<br>Score 2–6, 2.27 (1.74–2.96)
Y. Taniguchi, et al. 2015 [59]	773, Aged 65 or older living in the area who live in the town of Kusatsu	Prospective Cohort	2002-2007	3 years	Disability (All levels)	MMSE Annual Average Change	Age, sex, MMSE score, year of examination, grip strength, BMI, albumin level	(Ref: MMSE Annual Average Change 0)<br>0 to -5, 1.73 (0.93−3.23)<br>-5 to -1, 1.94 (1.01−3.45)<br>-1 to -2, 1.95 (1.02−3.76)<br>≤ -2, 3.16 (1.68−5.98)
H. Shimada, et al. 2019 [60]	4126, NCGG-SGS, Aged 65 years or older without a history of depression/ stroke/ Parkinson's disease/ dementia/ mini-mental state	Prospective Cohort	2016	49 months	Disability (All levels)	Psychological frailty: the co-presence of physical frailty and depressive mood (neither physical frailty nor depressive mood, phsical frailty only,	Age, sex, socioeconomic status, medication, smoking, living alone, primary diseases or geriatric syndromes (heart disease, pulmonary disease, hypertension,	(Ref: no physical frailty & depressive mood)<br>Physical frailty only, 1.69 (1.16–2.46)<br>Depressive mood only, 1.05 (0.79–1.39)<br>Psychological frailty, 2.24 (1.57–3.20)

	examination scores<18/ functional decline/ LTCI certification					depressive mood only, both (psychological frailty))	diabetes, osteoarthritis, and fall history), lifestyle activity (IADL, cognitive activity, social activity, and productive activity)	
Y. Tomata, et al. 2018 [61]	12365, The Ohsaki Cohort 2006, Aged 65 years or older in Ohsaki City	Prospective Cohort	2006	10 years	Disability (All levels)	Psychological distress as measured by K6	Age, sex, smoking, drinking, medical history, motor function, BMI, social support	Total participants<br>Psychological Distress Scale (K6 score)<br>(ref: <5)<br>5–9, 1.14 (1.04–1.22)<br>10–12, 1.28 (1.15–1.43)<br>≥13, 1.62 (1.44–1.84)<br>(*p* fore trend <0.001)
S. Yamazaki, et al. 2012 [62]	783, Aged 70 years or older	Prospective Cohort	2007	3 years	Disability (All levels)	GDS<br>(Depression group: GDS >=5, non-depression group: GDS <5)	Age, sex、living arrangement, hearing impairment, visual impairment, Chronic disease (hypertension, heart disease, osteoporosis, diabetes mellitus, stroke, mental disorder), Cognitive impairment, dietary variety score, IADL, falls experience during the past year, motor fitness scale,	(Ref: non-depression group)<br>Depression group, 2.34 (1.46–3.79)<br><br>Subgroup analysis<br>Depression group<br>Cognitive impairment, 3.51 (1.39–8.85)<br>MFS, 5.60 (1.32–23.81)<br>IADL, 3.37(1.65–6.85)<br><br>Non-depressed group<br>MFS, 2.97 (1.47–6.96)<br>Frequency of going out, 3.21 (1.47–6.96)

							frequency of going out (<1/w), self-rated health, social support	
K. Omori-Matsuda, et al. 2010 [63]	841, The Tsurugaya Project, Aged 70 years or older	Prospective Cohort	2003	4 years	Disability (All levels)	GDS<br>≥14 (or antidepressive agents use), 10-13, <9	Smoke, alcohol, medical history (stroke, myocardial infarction, cancer, deafness, cataract/glaucoma, arthritis), incontinence, social support, cognitive impairment (MMSE), physical function (time up and go test), serum albumin, spouse or not, socioeconomic status, BMI	GDS (Ref: <9)<br>Men<br>10–13, 1.31 (0.65–2.65)<br>≥14（or antidepressive agents), 2.19 (1.06 – 4.54)<br>(*p* for trend = 0.03)<br><br>Women<br>10–13, 1.31 (0.79–2.17),<br>≥14 (or antidepressive agents), 0.88 (0.50–1.54)<br>(*p* for trend = 0.71)
S. Bae, et al. 2018 [64]	4576, Aged 70 years or older living in Nagoya City	Prospective Cohort	2013	2 years	Disability (All levels)	Interaction of hearing impairment (Handicap Inventory for the Elderly and for Adults - Screening version (HHIE-S)) and social activity<br>(normal hearing and moderate to high social activity (reference)	Age, sex, smoking, medical history, frequency of exercise, MMSE score, socioeconomic status	(Ref: normal hearing and moderate to high social activity)<br>Hearing impairment only, 1.38 (1.07–1.77)<br>Low social activity only, 1.98 (1.26–3.11)<br>Hearing impairment and low social activity, 2.13 (1.31–3.45)

						low social activity only, hearing impairment only, hearing impairment and low social activity)		
K, Makino, et al, 2019 [65]	4365, NCGG-SGS, Aged 65 years or older	Prospective Cohort	2013	2 years	Disability (All levels)	Pain was assessed by face-to-face interviews<br>(1) Severity; no pain, mild, moderate, severe.<br>(2) Duration; chronic pain (pain persistent for at least 2 months) or non-chronic pain (pain persisting for less than 2 months)	Age, sex, medical condition (hypertension, diabetes mellitus, heart disease, osteoarthritis, and spinal diseases), polypharmacy (the number of medication use >=3/day), slow gait speed (<1.0 m/s), and depressive symptoms	(1) Severity (ref: no pain)<br>Severe pain, 1.66 (1.05–2.62)<br><br>(2) Duration<br>Not significant
M. Higashiguchi, et al. 2008 [66]	832, The Tsurugaya Project, Aged 70 years or older	Prospective Cohort	2003	3 years	Disability (All levels)	Serum albumin levels	Sex, age, marital status, social support, smoking, alcohol, depressive symptom (GDS), cognitive impairment (MMSE), medical history, self-rated health	(Ref: serum albumin level ≥4.4 g/dL)<br>≤3.8, 2.3 (1.2–4.5)<br>3.9–4.1, 1.8 (1.1–3.1)<br>4.2–4.3, 1.0 (0.6–1.8)<br>(p for trend = 0.001)
A. Hozawa, et al.2010 [67]	512, The Tsurugaya Project, Aged 70 years or older	Prospective Cohort	2002-2003	6 years	Disability (All levels)	Plasma NT-pro BNP levels	Age, sex, home blood pressure, smoking, serum creatinine, low serum	(Ref: plasma NT–proBNP level (pg/mL) of 25 percentile (< 47))<br>Disability

							albumin, low serum cholesterol, BMI, depressive symptoms, functional reach, history of cardiovascular disease	90 percentile (≥ 241), 2.04 (1.07–3.86)<br><br>Disability & death<br>90 percentile (≥ 241), 1.90 (1.06–3.39)
A. Hozawa, et al. 2012 [68]	505, The Tsurugaya Project, Aged 70 years or older	Prospective Cohort	2002-2003	6 years	Disability (All levels)	Serum adiponectin levels	Model 1: age, sex, smoking, alcohol, history of cardiovascular diseases, and metabolic syndrome-related factors. Model 7: Model 1 +NT-pro BNP, malnutrition, depression, eGFR, physical function	(Ref: serum adiponection levels (mg/L) of 2.0–7.9)<br>8.0–10.9, 1.33(0.78–2.25)<br>11.0–15.9, 1.09 (0.64–1.87)<br>16.0–22.3, 1.14 (0.61–2.11)<br>≥22.4, 1.41 (0.69–2.86)
A. Hozawa, et al. 2013 [69]	Case 165, Control 177, The Tsurugaya Project, Aged 70 years or older	Nested case-control	2002-2003	7 years	Disability (All levels)	Serum isoflavones: genistein, daidzein, glysitein, and equol	Age, sex, smoking, drinking, blood pressure, casual blood glucose, total cholesterol, serum albumin, functional reach, BMI, depressive symptom, stiffness of calcaneus, history of cardiovascular diseases, history of cancer, total energy intake, green tea consumption, meat	(Ref: serum equol levels (ng/mL) of ≤0.9)<br>≥23.6, 0.51 (0.27–0.96)

							consumption, and physical activity	
R. Hoshi, et al.2013 [70]	827, The Tsurugaya Project, Aged 70 years or older	Prospective Cohort	2003	6 years	Disability (All levels)	Serum total cholesterol levels	Sex, age, smoking, drinking, depressive symptom, socioeconomic status, cognitive function, physical function, BMI, serum albumin, use of use of hyperlipidemia medication, history of stroke, history of osteoporosis, Alanine aminotransferase (ALT), Aspartate aminotransferase (AST), history of liver diseases, history of cancer	(Ref: serum total cholesterol level (mg/dL) of 212–230)<br><177, 1.91 (1.23–2.98)<br>177–194, 1.36 (0.85–2.18)<br>195–211, 0.99 (0.62–1.56)<br>≥231, 1.38 (0.88–2.17)
K. Yamagishi, et al. 2014 [71]	Case 65, Control 130, CIRCS, Aged 40-69 years	Nested case-control	1984-1994	5 years	Dementia	(1) Serum coenzyme Q10 (grouped by quartile)<br>(2) Serum coenzyme Q10/total cholesterol ratio	BMI, smoking, alcohol, DBP, diabetes mellitus and medication of hypertension and hypercholesterolemia	(Ref: serum coenzyme Q10 levels (nmol/L) of 228–558)<br>559–765, OR=0.68 (0.26–1.78)<br>766–1015, OR=0.92 (0.33–2.56)<br>1016–2353, OR=0.23 (0.06–0.86)<br>(*p* for trend = 0.05)

								(Ref: serum coenzyme Q10/total cholesterol ratio of 54.1–107.5)<br>107.6–151.3, OR= 0.67 (0.25–1.78)<br>151.4–203.6, OR=0.73 (0.28–1.92)<br>203.7–489.1, OR=0.21 (0.05–0.90)<br>(*p* for trend = 0.04)
C. L. Chei, et al.2014 [72]	Case 275, Control 550, CIRCS, Aged 40-69 years	Nested case-control	1999	14 years	Dementia	(1) Serum high-sensitivity C-reactive protein(hs-CRP) levels (mg/L) grouped by quartile (0.002-0.016 (ref) , 0.017-0.041, 0.042-0.088, 0.090-3.11)<br>(2) 1 SD increment of log hs-CRP levels	SBP, antihypertensive medication use, borderline diabetes, diabetes, BMI, smoking, alcohol, serum total cholesterol levels	Total dementia<br>Not significant<br><br>Dementia with history of stroke<br>(1) (Ref: serum hs-CRP levels (mg/L) of 0.002–0.016)<br>0.017–0.041, 2.15 (0.90–5.15)<br>0.042–0.088, 2.06 (0.82–5.21)<br>0.090–3.11, 2.72 (1.12–6.64)<br><br>(2) 1SD increment of log hs-CRP levels, 1.35 (1.02 -1.79)<br><br>Dementia without history of stroke<br>Not significant
K. Yamagishi, et al. 2017 [73]	Case 315, Control 630, CIRCS, Aged 40-74 years	Nested case-control	1989-1991 and 1995 in Ikawa town	5 years and 14 years	Dementia	(1) Serum alpha-lineic acid (grouped by quartile)	Age, smoking, SBP, diabetes mellitus, medication of hypertension	(1) (Ref: serum alpha-linolenic acid (% total fatty acid) of 0.30-0.74)<br>0.75–0.90, OR=0.57 (0.39–0.85)

			and 1984-1994 in Kyowa town			(2) Serum eicosatetraenoic acid (grouped by quartile)<br>(3) Serum docosahexaenoic acid (grouped by quartile)		0.91–1.11, OR=0.51 (0.34–0.76)<br>1.12–2.26, OR=0.61 (0.41–0.90)<br>(*p* for trend = 0.01)<br><br>(2) (Ref: serum eicosatetraenoic acid (% total fatty acid) of 0.50–2.14)<br>2.15–3.23, OR=0.99 (0.67–1.49)<br>3.24–4.66, OR=0.90 (0.58–1.39)<br>4.67–12.8, OR=0.79 (0.49–1.26)<br>(*p* for trend = 0.28)<br><br>(3) (Ref: serum docosahexaenoic acid (% total fatty acid) of 1.78–4.19)<br>4.20–5.23, OR=0.88 (0.58–1.31)<br>5.24–6.52, OR=1.20 (0.77–1.86)<br>6.53–11.1, OR=1.18 (0.72–1.94)<br>(*p* for trend = 0.34)
T. Doi, et.al. 2016 [74]	4133, OSHPE, Aged 65 years or older	Prospective Cohort	2011-2012	29.2 months	Disability (All levels)	Serum IGF-1 levels (grouped by quartile)	Age, BMI, smoking, alcohol, medical history (hypertension, hyperlipidemia, and diabetes), socioeconomic status, sleep duration,	(Ref: serum IGF-1 levels (mg/mL) of ≥120)<br>≤82, 1.72 (1.06–2.81)<br>83–100, 1.64 (0.99– 2.71)<br>101–119, 1.31 (0.76–2.25)

							physical exercise, GDS, MMSE and gait speed	
H. Yamazaki, et al. 2019 [75]	2484, LOHAS, Aged 65 years or older	Prospective Cohort	2008-2010	5.75 years	Disability (Care-need level ≥3) or Death	Alanine aminotransferase (ALT) (<10, 10-20, 20-30, 30-40, ≥40 U/L)	Age, sex, BMI, smoking, alcohol, aspartate aminotransferase, physical activity, physical function, grip strength, and the number of comorbidities	(Ref: ALT (U/L) of 20–30)<br><10, 3.02 (1.57–5.81)<br>10–20, 1.55 (1.07–2.24)<br>30–40, 1.29 (0.72–2.31)<br>≥40, 1.49 (0.58–3.25)
S. Takahashi, et al. 2018 [76]	5755, The Iwate-KENCO study, Aged 65 years or older in Iwate	Prospective Cohort	2002-2005	5.7 years	Disability (All levels)	(1) Urinary albumin-creatinine ratio (UACR)<br>(2) Plasma B-type natriuretic peptide concentration (BNP)<br>(3) Serum high-sensitivity C-reactive protein concentration (hs-CRP)	Age, sex, BMI, SBP, DBP, TC, HDLC, Non-HDLC, HB, HbA1c, eGFR, socioeconomic status, smoking, alcohol, hypertension, diabetes mellitus, dyslipidemia, AF, interim CVD	UACR (ref: Q1)<br>Q2, 1.24 (0.98–1.57)<br>Q3, 1.36 (1.07–1.72)<br>Q4, 1.69 (1.35–2.12)<br><br>BNP (ref: Q1)<br>Q2, 0.95 (0.75–1.20)<br>Q3, 0.84 (0.67–1.05)<br>Q4, 1.04 (0.84–1.30)<br><br>hs–CRP (ref: Q1)<br>Q2, 0.92 (0.74–1.15)<br>Q3, 0.86 (0.69–1.07)<br>Q4, 0.93 (0.75–1.16)

T. Okuno, et al. 2017 [77]	1090, Aged 65-94 years in a town Ishikawa	Prospective Cohort	2008	5 years	Disability (All levels)	ECG abnormality by Minnesota code (Q-wave abnormality, QRS axis deviation, ST-T abnormality, atrioventricular conduction abnormality, intraventricular conduction defect, arrhythmia) Stratified by prior history of cardiovascular disease(s)	Age, sex, BMI, living alone, hypertension, diabetes, CKD, proteinuria, hyperuricemia, hypoalbuminemia, hypocholesteremia	Total population<br>Any major ECG abnormality, 1.76 (1.24–2.49)<br><br>Group with prior history of CVD<br>Any major ECG abnormality, 0.86 (0.44–1.69)<br><br>Group with no prior history of CVD<br>Any major ECG abnormality, 2.42 (1.58–3.69)
A. Honda, et al. 2014 [78]	1580, Aged 65 years or older	Prospective Cohort	2001	5.8 years	Disability (All levels)	Body mass index	Age, sex, hypertension, dyslipidemia, smoking, alcohol, monthly medical expenditure in the fiscal year 2001 using propensity scores	(Ref: BMI of 18.5- <25.0)<br>Young-old elderly (aged 65-74 years)<br>Men and women<br><18.5, 4.26 (1.69–10.72)<br>Men<br><18.5, 3.78 (1.05–13.54)<br>Women<br><18.5, 4.53 (1.16–17.66)<br><br>Old–old elderly (aged 75 years or older)<br>Not significant

S. Zhang, et al. 2016 [79]	12376, The Ohsaki Cohort 2006, Aged 65 years or older	Prospective Cohort	2006	5.7 years	Disability (All, due to arthralgia /fractures, due to stroke, due to dementia, due to other disease)	Body mass index (<21, 21-<23, 23-<25, 25-<27, 27-<29, ≥29 kg/m^2)	Age, sex, smoking, medical history (stroke, myocardial infarction, diabetes, digestive system diseases or cancer), socioeconomic status, MMSE score, depressive symptom	(Ref: BMI of 25–<27 kg/m^2)<br>Function disability<br><21, 1.56 (1.36–1.80)<br>21– <23, 1.23 (1.07–1.41)<br>23– <25, 1.04 (0.90–1.20)<br>27– <29, 1.04 (0.86–1.26)<br>≥29, 1.47 (1.20–1.80)<br><br>Functional disability due to dementia<br><21, 2.48 (1.70–2.69)<br>21–<23, 2.25 (1.54–3.27)<br>23–<25,1.17 (,0.78–1.77)<br>27–<29, 0.84 (0.47–1.52)<br>≥29, 1.25 (0.67–2.33)<br><br>Functional disability due to stroke<br><21, 1.22 (0.86–1.73)<br>21– <23, 1.02 (0.72–1.44)<br>23– <25, 0.97 (0.69–1.37)<br>27– <29, 1.07 (0.69–1.68)<br>≥29, 0.85 (0.47–1.53)<br><br>Functional disability due to joint disease<br><21, 0.91 (0.51–1.20)<br>21– <23, 0.97 (0.68–1.38)

								23– <25, 0.77 (0.53–1.12)<br>27– <29, 0.87 (0.54–1.41)<br>≥29, 2.17 (1.40–3.35)
S. Zhang, et al.2019 [80]	12666, The Ohsaki Cohort 2006, Aged 65 years or older	Prospective Cohort	2006	10 years	Disability (All levels)	Body mass index (<19, 19-<21, 21-<23, 23-<25, 25-<27, 27-<29, ≥29 kg/m^2)	Sex, smoking, alcohol, medical history (stroke, myocardial infarction, digestive system diseases, or cancer), motor function score, psychological distress, pain, socioeconomic status, participation in community activities (activities in neighborhood association, sports or exercise, volunteering and social gathering)	(Ref: BMI of 25–27 kg/m^2)<br><19, 1.63 (1.46–1.81)<br>19– < 21, 1.37 (1.25–1.51)<br>21– < 23, 1.23 (1.13–1.34)<br>23– < 25, 1.07 (0.98–1.17)<br>27– < 29, 1.11 (0.99–1.24)<br>≥29, 1.31 (1.15–1.48)<br><br>Disability-free survival (50th percentile differences in months in age at disability or death)<br>(Ref: BMI of 25−27 kg/m^2)<br><19, -20.8 (-26.4, -15.2)<br>19- < 21, -13.5 (-18.2, --8.7)<br>21- < 23, -9.8 (-14.2, -5.4)<br>23- < 25, -2.9 (-7.5, 1.7)<br>27- < 29, -2.7 (-8.4, 2.9)<br>≥29, -11.5 (-19.6, -3.5)
T.Ashida,et al. 2016 [81]	12991, JAGES, Aged 65 years or older	Prospective Cohort	2003	4 years	Disability (All levels)	Participation in group activities<br>(1) Sports group	Age, marital status, employment status, three major diseases (cancer,	Men<br>(1) Sports group, 0.66 (0.51–0.85)<br>(2) Hobby group, 0.69 (0.55–0.87)

						(2) Hobby group<br>(3) Volunteer group<br>(4) Facilitator role	heart disease, and stroke), municipality	(3) Volunteer group, 0.81 (0.57–1.15)<br>(4) Have facilitator role, 0.82 (0.66–1.02)<br><br>Women<br>(1) Sports group, 0.58 (0.44–0.76)<br>(2) Hobby group, 0.69 (0.55–0.80)<br>(3) Volunteer group, 0.86 (0.62–1.19)<br>(4) Have facilitator role, 0.70 (0.56–0.88)
T. Otsuka, et al. 2018 [82]	11992, The Ohsaki Cohort 2006, Aged 65 years or older	Prospective Cohort	2006	8 years	Disability (All levels)	social participation<br>(1) Volunteering<br>(2) Hobby activities<br>(3) Activities in neighborhood association<br>(4) Number of social participation type	Age, sex, medical history (stroke, hypertension, myocardial infarction, arthritis, osteoporosis, fracture, cancer), socioeconomic status, smoking, alcohol, BMI, motor function score, cognitive impairment, pain, employment, and frequency of three kinds of social participations	Frequency of social participation (ref=non)<br>(1) Volunteering<br><1 time per month, 0.87 (0.78–0.97)<br>1–3 times per month, 0.92 (0.80–1.05)<br>≥1 time per week, 0.87 (0.72–1.05)<br><br>(2) Hobby activities<br><1 time per month, 0.85 (0.75–0.95)<br>1–3 times per month, 0.85 (0.77–0.94)<br>≥1 time per week, 0.77 (0.69–0.86)<br><br>(3) Activities in neighborhood association<br><1 time per month, 0.96 (0.87–1.05)<br>1–3 times per month, 0.92 (0.83–1.01)<br>≥1 time per week, 1.14 (0.96–1.35)

								(4) Number of social participation type<br>One activity, 0.85 (0.78–0.94)<br>Two activities, 0.86 (0.78–0.95)<br>Three activities, 0.75 (0.68–0.84)
Y. Nemoto, et al. 2017 [83]	13580, AGES, Aged 65 years or older	Prospective Cohort	2003	7.9 years	Dementia	(1) Social participation<br>(2) Leadership position on organization	Age, sex, smoking, alcohol, socioeconomic status, marital status, living arrangement, occupational status, walking time, medical history, depressive symptom, IADL	(1) Social participation (ref: non-participants)<br>Young-old<br>Participants, 0.75 (0.64–0.88)<br><br>Old–old<br>Participants, 0.91 (0.81–1.03)<br><br>(2) Leadership position on organization (ref: regular-members)<br>Young-old<br>Non–participants, 1.22 (1.02–1.46)<br>Participants, 0.81 (0.65–0.999)<br><br>Old–old<br>Non–participants, 0.99 (0.86–1.13)<br>Participants, 0.98 (0.83–1.14)
T. Saito, et al. 2018 [84]	13468, AGES, Aged 65 years or older	Prospective Cohort	2003	3436 days (9.4 years)	Dementia	Social network (marital status, contact with relatives and contact with	Age, sex, socioeconomic status, depressive symptom, cognitive	(ref= no)<br>Married, 0.88 (0.79–0.99)<br>Contact (relatives), 0.89 (0.76–1.05)

						friends), social support (family member/ relative/ friend/ neighbor), social activities (group participation, work engagement)	impairment, IADL, stroke, diabetes, daily walking time and hobbies	Contact (friends), 0.83 (0.73–0.94)<br>Group participation,0.89 (0.80–0.98)<br>Work engagement, 0.88 (0.77–0.99)<br>Support (family), 0.88 (0.79–0.99)<br>Support (relatives), 1.03 (0.95–1.13)<br>Support (friends), 0.98 (0.89–1.08)<br><br>Diverse social relationship engagement score (ref: 0 or 1)<br>Score 2, 0.86<br>Score 3, 0.75<br>Score 4, 0.65<br>Score 5, 0.54<br>(*p* for trend <0.001)
H. Yokokawa, et al. 2009 [85]	12056, The Iwate-KENCO study, Aged 65 years or older living in Iwate	Prospective Cohort	2002-2005	2.7 years	Disability (All levels)	homebound status (walking outdoors <5 minutes/day)	Age, SBP, BMI, HbA1c, total cholesterol, HDL-cholesterol, smoking, alcohol, exercise habit, regular job	(Ref: not being homebound)<br>Men<br>Being homebound, 1.07 (0.76–1.52)<br><br>Women<br>Being homebound, 1.64 (1.29–2.09)
H. Makizako, et al. 2015 [86]	4304, OSHPE, Aged 65 years of older	Prospective Cohort	2011-2012	2 years	Disability (All levels)	Social frailty (going out less frequency compared with last year, visiting friends sometimes, feeling	Age, sex, BMI, MMSE, number of prescribed medications, hypertension, heart disease, diabetes,	(Ref: nonfrail)<br>Prefrail, 1.53 (1.02–2.31)<br>Frail, 1.66 (1.00–2.74)

						helpful to friends or family, living alone, and talking with someone every day; 1 items criterion response = prefrail, 2 or more criteria responses = frail)	osteoporosis, GDS and physical frailty	
T. Noguchi, et al. 2019 [87]	73021, JAGES, Aged 65 years or older	Prospective Cohort	2010-2012	3 years	Disability (Care-need level ≥2)	Community social capital<br>(1) Community civic participation<br>(2) Social cohesion<br>(3) Reciprocity	Age, socioeconomic status , marital status, self-rated health, self-reported BMI, IADL, present illness, depressive symptom, lifestyle (smoking history, alcohol consumption and frequency of going outside) and individual social components (urbanization)	Community-level social capital (1 SD increase)<br>Men<br>Civic participation, 0.972 (0.893–1.058)<br>Social cohesion, 0.910 (0.830–0.998)<br>Reciprocity, 0.920 (0.829–1.021)<br><br>Women<br>Civic participation, 0.999 (0.918–1.087)<br>Social cohesion, 0.930 (0.847–1.020)<br>Reciprocity, 1.002 (0.901–1.114)
E. Saito, et al. 2014 [88]	1347, aged 70 years or older	Prospective Cohort	2004 and 2005	2316.3 days	Disability (All levels)	Household condition	Age and sex	(Ref. three-generation)<br>Children-only, 1.61 (1.08−2.40)<br>Spouse-only, 0.91 (0.56−1.48)<br>Living alone, 1.13 (0.86−1.48)

T. Saito, et al. 2017 [89]	6600, AGES, Aged 65 years or older	Prospective Cohort	2003	9.4 years	Disability (Care-need level ≥2)	Living arrangements (with spouse, with non-spousal cohabitants, and living alone)	Age, socioeconomic status, self-rated health, presence of illness, depressive symptom, IADL, cognitive impairment, BMI, smoking, alcohol, daily walking time, emotional support received, emotional support provided, instrumental support received, instrumental support provided	(Ref: living with spouse)<br>Men<br>Living with non-spousal cohabitants, 1.31 (1.10–1.56)<br>Living alone, 1.35 (1.03–1.77)<br><br>Women<br>Living with non–spousal cohabitants, 1.08 (0.94–1.24)<br>Living alone, 1.16 (0.98–1.38)
R. Momosaki, et al. 2019 [90]	31273, JAGES, Aged 65 years or older	Prospective Cohort	2010-2012	6 years	Disability (All levels)	Subjective food store availability (evaluated by a self-reported questionnaire)<br>Objective food store availability (evaluated by geographic information system, high= had stores along a straight line within a radius of 500 m from the center of their residential	Age, sex, socioeconomic status (education attainment, marital status, employment status, yearly income, living situation), environmental status (driving status, neighborly relationships population density), walking and going out, dietary food intake, BMI, and	(1) Subjective food store availability (ref: high)<br>Low, 1.18 (1.11–1.25)<br>(2) Objective food store availability (ref: high)<br>Low, 1.00 (0.94–1.08)

						community blocks, low= did not have stores along a straight line within a radius of 500 m from the center of their residential community blocks)	comorbidities (malignancy, heart disease, stroke, hypertension, diabetes, joint disease/ neuralgia, traumatic fracture, respiratory disease, gastrointestinal disease, dyspnea, depression, cognitive decline)	
Y. Tani, et al. 2019 [91]	49511, JAGES, Aged 65 years or older	Prospective Cohort	2010-2012	3 years	Dementia	Availability of food stores The number of food stores selling fruits and vegetables with 500 meters or 1 kilometer of residence assessed by objective (Geographical Information System based) and subjective (participant-reported) measurements.	Age, sex, socioeconomic status, marital status, employment status, driving status, public transportation, prefecture of residence, BMI, frequency of vegetable/fruit intake, walking time and frequency of going out, hypertension, diabetes, hearing loss, depressive symptom, IADL, cognitive impairment, population	Availability food stores with 500 meters of residence by GIS<br>(Ref: Q4)<br>Q3, 1.19 (1.06–1.34)<br>Q2, 1.20 (1.07–1.35)<br>Q1, 0.98 (0.87–1.11)<br><br>Availability food stores with 1 kilometer of residence by GIS<br>(Ref: Q4)<br>Q3, 1.34 (1.18–1.53)<br>Q2, 1.41 (1.23–1.61)<br>Q1, 1.16 (1.00–1.34)

							density of inhabitable area of residence	Subjective availability food stores (Ref: Q4)<br>Q3, 1.10 (0.98–1.23)<br>Q2, 1.19 (1.04–1.35)<br>Q1, 1.30 (1.10–1.52)
T. Takasugi, et al. 2019 [92]	52063, JAGES, Aged 65 years or older	Prospective Cohort	2010-2012	6 years	Dementia	Socioeconomic status<br>(1) Educational attainment<br>(2) Longest job held<br>(3) Equivalized household income	Age, sex, marital status, residential status, , current occupational status, smoking, alcohol, daily walking time, medical history (stroke, hypertension, and diabetes) and depression symptom (GDS)	(1) Education attainment (ref: ≥13 years)<br>Men<br><6 years, 1.34 (1.04–1.73)<br>6–9 years, 1.05 (0.93–1.19)<br>10–12 years, 1.03 (0.90–1.17)<br><br>Women<br><6 years, 1.21 (1.00–1.45)<br>6–9 years, 1.00 (0.87–1.15)<br>10–12 years, 0.96 (0.83–1.11)<br><br>(2) Longest job held (ref: professional/ technical)<br>Men<br>Administrative, 0.86 (0.72-1.03)<br>Clerical, 1.08 (0.91-1.27)<br>Sales/service, 0.88 (0.75-1.04)<br>Skilled/labour, 0.87 (0.75-1.00)

								Agriculture/ forestry/ fishery, 0.96 (0.83-1.12)<br>Others, 1.09 (0.93-1.28)<br>No occupation, 1.25 (0.99-1.58)<br><br>Women<br>Administrative, 0.88 (0.54-1.42)<br>Clerical, 0.91 (0.76-1.09)<br>Sales/ service, 0.92 (0.76-1.11)<br>Skilled/ labour, 0.97 (0.80-1.18)<br>Agriculture/ forestry/ fishery, 1.03 (0.86-1.25)<br>Others, 0.98 (0.83-1.16)<br>No occupation, 0.95 (0.80–1.13)<br><br>(3) Equivalized household income (ref: ≥4000000)<br>Men<br><1999999, 0.97 (0.83–1.13)<br>2000000–3999999, 0.95 (0.81–1.11)<br><br>Women<br><1999999, 0.83 (0.72–0.96)<br>2000000–3999999, 0.85 (0.74–0.99)

D. Nurrika, et al. 2019 [93]	8680, The Ohsaki Cohort 2006, Aged 65 years or older	Prospective Cohort	2006	9 years	Disability (All levels)	Education level (below upper secondary education (left school <16 years old, reference), upper secondary education and above (left school ≥16 years old)	Age, sex, smoking, alcohol, social support, community activities	(Ref: below upper secondary education)<br>Upper secondary education and above, 0.81 (0.75–0.88)<br><br>Age 65–74 years<br>Upper secondary education and above, 0.62 (0.54–0.72)<br><br>Age ≥75 years<br>Upper secondary education and above, 0.91 (0.83–1.00)
N. Kondo, et al. 2009 [94]	7673, JAGES, Aged 65 years or older	Prospective Cohort	2003	3 years	Disability (All levels)	Relative deprivation (Yitzhaki index)	Age, socioeconomic status, marital status, medical care utilization	Men<br>HR for disability per 1SD increase in relative deprivation,<br>Reference group defined by<br>Area; 1.14 (1.00–1.30)<br>Age; 1.14 (1.00–1.31)<br>Education; 1.15 (1.01–1.31)<br>Area and sex; 1.14 (1.00–1.30)<br>Age and sex; 1.14 (1.00–1.31)<br>Education and sex; 1.15 (1.01–1.31)<br>Area, sex and age; 1.13 (0.99–1.29)<br>Area, sex and education; 1.14 (1.01–1.30)

								Women<br>HR for disability per 1SD increase in relative deprivation,<br>Reference group defined by<br>Age; 1.18 (1.00–1.39)<br><br>Additional adjustment for lifestyle factors attenuated the hazard ratios to statistical non-significance.
N. Kondo, et al.2012 [95]	583, Y-HALE, Aged 65 years or older	Prospective Cohort	2003	8 years	Disability (Care-need level ≥3)	Rotating savings and credit association (*Mujin* in Japanese) (2 components) intensity and attitude financing	Age, sex, having spouse, household members physical health (Physical Component Summary of the Medical Outcomes Study Short Form-36), socioeconomic status, social activity	Intensity and attitude (per 1 SD), 1.01 (0.81–1.25)<br>Financing (per 1 SD), 1.20 (1.07–1.35)
Y. Tomata, et al. 2011 [96]	14636, The Ohsaki Cohort 2006, Aged 65 years or older	Prospective Cohort	2006	1 years	Disability (All levels)	The Kihon Checklist	Age, sex	The Kihon Checklist ≥10 items; OR= 6.54 (5.31–8.04), AUC = 0.83<br>Prevention program candidate; OR= 3.80 (3.02–4.78), AUC = NA
T. Kamegaya, et al. 2017 [97]	21325, Aged 65 years or older	Prospective Cohort	2010-2011	3 years	Disability (All levels)	(1) Total score of first 20 items of Kihon Checklist (KCL)	Model 1; age and sex,<br>Model 2; age, sex, physical function, nutritional	Model 1<br>(1) Total score of first 20 items of KCL, ≥10 items: OR=2.71 (2.22–3.32)

						(2) Physical function<br>(3) Nutritional condition<br>(4) Oral function<br>(5) Selected as a care prevention program candidate (if the subject meets any of criteria mentioned in (1)-(4))<br>(5) Homebound state (items number 16-17)<br>(6) Dementia (items number 18-20)<br>(7) Depression (items number 21-25)	condition, oral function, homebound state, dementia, depression	(2) Physical function (items number 6–10), ≥3 items: OR=2.29 (2.05–2.55)<br>(3) Nutritional condition (items number 11–12), 2 items: OR=1.85 (1.38–2.48)<br>(4) Oral function (items number 13–15), ≥2 items: OR=1.40 (1.25–1.57)<br>(5) Selected as a care prevention program candidate: OR=1.90 (1.73–2.08)<br>(6) Housebound state (items number 16–17), Care projects candidates who checked item number 16: OR=1.91 (1.55–2.37)<br>(7) Dementia (items number 18–20), Care projects candidates who checked ≥1 items: OR=1.97 (1.75–2.20)<br>(8) Depression (items number 21–25), ≥2 items: OR=1.96 (1.73–2.22)<br><br>Model 2<br>(2) Physical function (items number 6–10), ≥3 items: OR=1.87 (1.65–2.13)<br>(3) Nutritional condition (items number 11–12), 2 items: OR=1.60 (1.18–2.16)<br>(6) Dementia (items number 18–20), Care projects candidates who checked ≥1 items: OR=1.30 (1.11–1.51)

								(7) Depression (items number 21–25) ≥2 items: OR=1.24 (1.06–1.44)
D. Okabe, et al. 2018 [98]	9756, JAGES, Aged 65 years or older	Prospective Cohort	2010	3 years	Disability (Care-need level ≥2) or Death	The Kihon Checklist	Age, sex, smoking, alcohol, socioeconomic status	The Kihon Checklist<br>Total score of first 20 items of the Kihon checklist (KCL) ≥10 items, 3.63 (2.54–5.21)<br>Physical function (items No. 6–10) ≥3 items, 2.20 (1.72–2.80)<br>Nutrition condition (items No. 11–12) 2 items, 3.16 (2.05–4.88)<br>Housebound (items No.16–17) 2 items, 1.72 (1.37–2.15)<br>Dementia, (items No.18−20) ≥1 item, 1.44 (1.16-1.78)<br>Depression, (items No.21−25) ≥2 items, 1.95 (1.54−2.46)<br><br>Specific health checkups items<br>BMI (ref: 18.5- < 25 kg/m^2)<br>≥25 kg/m^2; 2.03 (1.40−2.95)<br>HDL-cholesterol (ref: ≥40 mg/dL)<br><40 mg/dL, 1.72 (1.29−2.29)<br>Aspartate aminotransferase (AST) (ref: 10−36 IU/L)

								<10 U/L or >=36 IU/L, 1.76 (1.24−2.50)<br>Fasting blood glucose (ref: <110 mg/dL),<br>≥110 mg/dL, 1.57 (1.13−2.18)<br>HbA1c (NGSP) (ref: <6.0%)<br>≥6.0 mg/dL, 1.37 (1.06−1.78)<br>Urine protein (ref: absence) Presence, 2.07 (1.56−2.76)
S. Satake, et al. 2017 [99]	5542, Aged 65 years or older in Higashi-ura town	Prospective Cohort	2010	3 years	Disability (All levels)	Three groups based on the Kihon Checklist score<br>Robust, 0-3<br>Pre-frail, 4-7<br>Frail, ≥8	Age, sex	(Ref: robust (0−3 of total score of the Kihon Checklist))<br>Prefrail (4−7), 2.027 (1.575−2.608)<br>Frail (≥8), 4.768 (3.733−6.089)
T. Katsura, et al. 2017 [100]	7820, Aged 65 years or older who lived Uji city	Retrospective Cohort	2008	5 years	Disability (All levels)	The Kihon Checklist	Age, sex, settlement, care prevention service, specific health checkup items and Kihon Checklist	Total<br>Abnormal liver function, 1.27 (1.08−1.50)<br>Anemia, 1.17 (1.02−1.34)<br>Urinary protein, 1.18 (1.01−1.38)<br>Presence of subjective symptom, 1.24 (1.08−1.43)<br><br>The Kihon Checklist<br>Inability to climb stairs without holding on to handrails or walls, 1.50 (1.29−1.74)<br>Going out less frequency in comparison with 6 months prior, 1.39 (1.20−1.62)

								Inability to enjoy what used to be fun, in the past 2 weeks, 1.37 (1.10−1.69)<br>The use of support for standing up after being seated on a chair, 1.30 (1.10−1.54)<br>Inability to travel on a bus or train alone, 1.30 (1.02−1.64)<br>Underweight, 1.26 (1.02−1.55)<br>Having high levels anxiety about falling down, 1.23 (1.06-1.42)<br>Having fallen down in the past year, 1.18 (1.02−1.38)
S. Shinkai, et al. 2010 [101]	916, Aged 75 years or older living in Kusatsu town Gunma	Prospective Cohort	2001	4 years	Disability (All levels)	Total score of the Kihon checklist	Age, sex, IADL (Instrumental Self-Maintenance subscale of TMIF-IC)	An increase of 1 point; OR = 1.21 (1.10−1.33)
Y. Kotaki, et al. 2019 [102]	8563, The Ohsaki Cohort 2006, Aged 65 years or older	Prospective Cohort	2006	5.7 years	Dementia	Total number of risk factors (diabetes mellitus, hypertension, obesity, physical inactivity, severe psychological distress, smoking and low educational attainment)	Age, sex	(Ref: no risk factors)<br>1 risk factor, 1.24 (0.92–1.70)<br>2 risk factors, 1.59 (1.18–2.15)<br>≥3 risk factors, 2.21 (1.62–3.01)

* Results show the hazard ratios. Indices other than the hazard ratio are indicated in the table.

Abbreviations in project names, AGES: Aichi Gerontological Evaluation Study, CIRCS: Circulatory Risk in Communities Study, JAGES: Japan Gerontological Evaluation Study, J-MACC: Japan Multicenter, Aging Cohort for Care Prevention, LOHAS: Locomotive Syndrome and Health Outcomes in the Aizu Cohort Study, OSHPE: Obu Study of Health Promotion for the Elderly, ROAD: Research on Osteoarthritis/ Osteoporosis

Against Disability study, The Iwate-KENCO study: Iwate Kenpoku (Northern part of prefecture in Japanese) Cohort Study, NCGG-SGS: The National Center for Geriatrics and Gerontology Study of Geriatric Syndromes, NISSIN: The New Integrated Suburban Seniority Investigation Project, Y-HALE: The Yamanashi Healthy-Active Life Expectancy (Y-HALE) study.

Abbreviations in variables, results: ADL: Activity of Daily Living, BMI: Body Mass Index, CKD: Chronic Kidney Disease, DBP: Diastolic Blood Pressure, ECG: Electrocardiogram, eGFR: estimated glemerular filtration rate, GDS: Geriatric Depression Scale, HbA1c: Glycated Haemoglobin, HR: Hazard ratio, IADL: Instrumental Activity of Daily Living, K6: the Kessler 6 scale, METS: Metabolic Equivalent of Task, MMSE: Mini Mental State Examination, NT-pro BNP: The N-terminal prohormone of brain natriuretic peptide, OR: Odds Ratio, SBP: Systolic Blood Pressure, SD: Standard Deviation, TC: Total Cholesterol.
